# The Relationship between Sarcopenic Obesity, Weight-Loss and Maintenance Outcomes during Obesity Management: Are Additional Strategies Required?

**DOI:** 10.3390/clinpract11030069

**Published:** 2021-08-18

**Authors:** Dana El Masri, Leila Itani, Hana Tannir, Dima Kreidieh, Marwan El Ghoch

**Affiliations:** Department of Nutrition and Dietetics, Faculty of Health Sciences, Beirut Arab University, P.O. Box 11-5020 Riad El Solh, Beirut 11072809, Lebanon; dana.masri@bau.edu.lb (D.E.M.); l.itani@bau.edu.lb (L.I.); hana.tannir@bau.edu.lb (H.T.); d.kraydeyeh@bau.edu.lb (D.K.)

**Keywords:** BMI, obesity, sarcopenic obesity, weight-loss percentage, clinical outcome, weight-loss maintenance

## Abstract

The lack of long-term maintenance of the weight loss achieved during weight-management programs is the major cause of failure in obesity treatments. The identification of factors related to this outcome has clinical implications. Therefore, we aimed to assess the relationship between sarcopenic obesity (SO) and the weight-loss percentage (WL%). The WL% was measured at the six-month follow-up and after more than 12 months, in 46 adult participants with obesity, during an individualized weight-management program where participants were categorized as having or not having SO at the baseline. At the six-month follow-up, participants with SO did not display a significant difference in terms of WL%, when compared to those without SO (−10.49 ± 5.75% vs. −12.73 ± 4.30%; *p* = 0.148). However, after a longer term (i.e., >12 months), the WL% appeared to be significantly lower in the former (SO vs. non-SO) (−7.34 ± 6.29% vs. −11.43 ± 4.31%; *p* = 0.024). In fact, partial correlation analysis revealed a relationship between SO at the baseline and a lower WL% after more than 12 months (*ρ* = −0.425, *p* = 0.009), after controlling for age, sex, and body mass index (BMI). Participants with SO appeared to face more difficulties in maintaining the achieved WL over a longer term (>12 months follow-up) by comparison with their counterparts (i.e., non-SO). Should this finding be replicated in larger-sample studies, new strategies should be adopted for these patients in order to improve this clinical outcome, especially during the weight-maintenance phase.

## 1. Introduction

Recently, a new phenotype, sarcopenic obesity (SO) [1]—which is represented by the coexistence of obesity (i.e., an increase in body fat mass deposition) and sarcopenia (i.e., a decrease in muscle mass and strength) [2,3,4]—was declared a priority for both researchers and clinicians by international scientific bodies, such as the European Society for Clinical Nutrition and Metabolism (ESPEN) and the European Association for the Study of Obesity (EASO) [5], since the presence of SO seems to be associated with several medical and psychosocial comorbidities [6,7,8,9,10,11,12], as well as a higher risk of mortality [13]. 

In addition, recent evidence has shown that SO is also associated with reduced energy expenditure (i.e., resting energy expenditure) [14], poor physical performance [15], and a more sedentary lifestyle (i.e., daily steps) [16]. This has opened up new directions in research, which aim to determine whether these metabolic and lifestyle disadvantages among participants with SO may have, in some way, a negative impact on weight-management outcomes, such as a lower rate of weight loss (WL) or more difficulties in weight maintenance at the long-term follow-up stage. This is particularly the case in the light of very recent findings that showed that during a lifestyle modification weight-management program for obesity, the presence of SO at the baseline increases the risk of early interruption of the treatment (i.e., the risk of an early drop-out at around six months) [17]. Despite the fact that an earlier study found that WL rates at three, six, and 12 months of follow-up after bariatric surgery did not differ among patients with SO when compared to those without SO [18], no data are yet available on WL outcomes determined by means of lifestyle modification programs, or with a follow-up that exceeds 12 months. 

That being said, there is a consensus that the main challenge of obesity treatment is not WL but rather weight maintenance over a longer period [19]; the inability to maintain weight represents the major cause of failure of weight-management programs associated with obesity [20]. In fact, approximately 30–35% of the lost weight is regained one year following treatment, and 50% of patients usually return to their baseline weight between three and five years after the WL [21]. 

Several factors (environmental, biological, behavioral, and others) have been identified as being related/associated with better/worse WL maintenance [22]. However, WL maintenance following any obesity treatment is complex and has not been fully understood [23]. Therefore, the identification of new factors that lead to the lack of long-term WL maintenance and the implementation of effective strategies to prevent weight regain are required; this is considered a key factor in ensuring the real success of weight-management treatments in relation to obesity.

In light of these considerations, the current study aimed to investigate the relationship between SO at the baseline, and WL and WL maintenance at the six-month follow-up and after more than 12 months in a “real-world” clinical setting involving treatment-seeking patients with obesity. Our hypothesis sought to find a relationship between the presence of SO and lower rates of WL and WL maintenance. 

## 2. Materials and Methods

### 2.1. Participant and Study Design

Forty-six participants seeking WL treatment were recruited consecutively, referred by general practitioners to the Nutritional and Weight Management Outpatient Clinic in the Department of Nutrition and Dietetics at Beirut Arab University (BAU) in Lebanon, between May 2017 and September 2020. Patients were considered eligible if they were aged 18 years or older, had a BMI equal to or greater than 30.0 Kg/m^2^, were identified as suitable for WL treatment, and had started the treatment successfully. The patients assessed for eligibility were included as they met the following inclusion criteria: they (i) were effectively enrolled on the program and (ii) had completed the WL phase of the treatment (at the six-month follow-up); they also had at least another six months of WL maintenance, followed up for a total of 12 months or more. The exclusion criteria were being pregnant or lactating (i.e., in females), taking medications affecting body composition or weight, or having medical comorbidities associated with weight loss (i.e., cancer) and/or severe (uncontrolled) psychiatric disorders (acute psychotic states, bipolar disorder, etc.). 

### 2.2. Weight-Management Program Description

The program is based on a low-calorie diet, and the protocol for the treatment essentially involved a personalized, cognitive, behavioral treatment program, designed for patients with obesity; its details have been described elsewhere [24,25]. Patients were instructed to follow a low-energy diet (females 1000–1200 kcal/day and males 1500–1800 kcal/day) based on the Mediterranean diet. The program lasted 18 months and comprised one to two sessions in a preparatory phase, followed by two main phases. Phase 1 (the weight-loss phase) lasted six months, and phase 2 (the weight-maintenance phase) lasted 12 months with monthly sessions [25]. Each session lasted for 30 min, during which the patient received the six modules of the program: (1) monitoring food intake (i.e., a real-time monitoring sheet), (2) changing eating habits, (3) developing an active lifestyle (i.e., 10,000 daily steps), (4) addressing obstacles to weight loss, (5) addressing weight loss and primary goals, and (6) addressing obstacles to weight maintenance [25].

All procedures performed in studies involving human participants were in accordance with the ethical standards of the BAU research ethics committee (2017H-0035-HS-R-0242) and the 1964 Helsinki Declaration and its subsequent amendments or comparable ethical standards. The study was approved by the Institutional Review Board of BAU, and all participants provided informed, written consent. 

### 2.3. Demographics and Clinical Status

Participants were required to complete a questionnaire in order to retrieve information regarding their medical history, as well as social demographic and clinical status (age, sex, marital status, employment, level of education, etc.).

### 2.4. Baseline Measures

Body weight and height were measured using an electronic weighing scale (SECA 2730-ASTRA, Hamburg, Germany) and a stadiometer. The participants’ BMI was then calculated according to the standard formula of body weight in kilograms divided by the square of the height in meters. 

Body composition was measured by means of a segmental bioelectrical impedance analyzer (MC-780MA, Tanita Corp., Tokyo, Japan) [26]. After the gender, age, and height information had been entered into the machine, the participants were invited to stand in a stable position, in bare feet. The device provided separate body mass readings for different segments of the body, using an algorithm incorporating impedance, age, and height, to estimate the total and regional fat mass (FM) and fat-free mass (FFM) [26,27,28]. The SO was defined, based on the definition of Oh and colleagues, that is, a score of less than 23.4 in females and less than 29.6 in males, using the following formula: (appendicular skeletal muscle mass (ASM)/weight) × 100% [29]. ASM was defined as the sum of muscle mass in both the arms and legs.

Obesity comorbidities in this study were defined as the presence of any diseases, such as type 2 diabetes, cardiovascular diseases (coronary heart disease, stroke, transient ischemic attack, or peripheral arterial disease), or dyslipidemia (a decreased concentration of high-density lipoprotein cholesterol and an increased concentration of high-density lipoprotein cholesterol and triglycerides), based on self-reported diagnosis, either simultaneously or separately.

### 2.5. Follow-Up Measures

Body weight was measured and WL% was determined at the six-month follow-up and after more than 12 months by analyzing the medical records, taking into account the date of the visit when patients were last seen.

### 2.6. Statistical Analysis

Normality was checked using Shapiro–Wilk and Kolmogorov–Smirnov tests. The data are presented as mean and standard deviation for continuous variables. Categorical variables are presented as frequencies and proportions. A student’s *t*-test was used for mean comparison. A chi-squared test was used to compare frequencies. A paired t-test was conducted to compare body weight WL and WL% at the six-month follow-up and after more than 12 months of treatment. A partial correlation analysis was conducted to study the association between SO and the WL% among the study participants. All analysis was conducted using SPSS 26.0, and all significance was considered at *p* < 0.05.

## 3. Results

The characteristics of the study sample at baseline are presented in Table 1. The study sample comprised 46 participants who had completed six months of WL, followed up after at least 12 months (WL maintenance phase) and included 10 males (21.7%) and 36 females (78.3%). The mean age was 44.5 ± 15.85 years. SO affected 21 patients (45.7%), mostly females (76.2%). The participants’ distribution across categories of sociodemographic characteristics at baseline did not differ among those with or without SO. Likewise, the distribution of obesity comorbidities was similar among those with or without SO.

As for anthropometric characteristics and body composition at baseline, significant differences were observed between the categories of SO. Participants with SO had a significantly higher body weight (97.77 ± 14.88 vs. 87.32 ± 13.70 Kg, *p* = 0.018), BMI (38.64 ± 4.55 vs. 33.25 ± 3.59 Kg/m^2^, *p* < 0.0001), WC (115.31 ± 12.89 vs. 107.14 ± 10.87 cm, *p* = 0.047), BF (45.04 ± 12.99 vs. 32.16 ± 5.34 Kg, *p* < 0.0001), and BF% (44.63 ± 6.08% vs. 37.07 ± 4.02%, *p* < 0.0001). 

**Figure 1:**
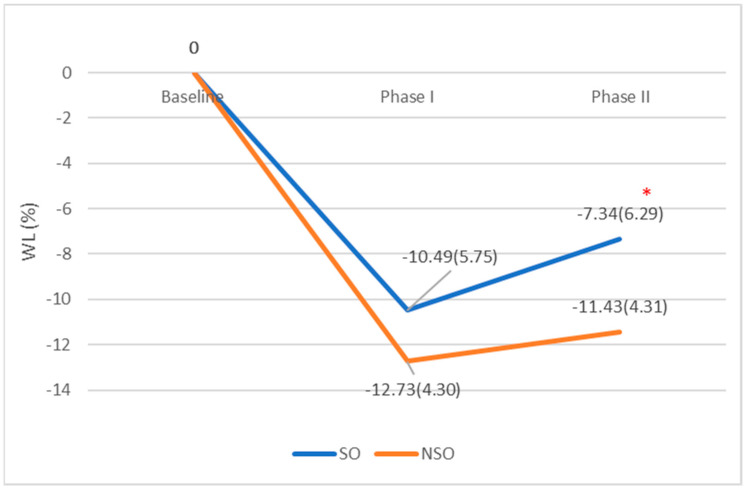
Mean WL% at 6-month and >12-month follow-up. * Significantly different then Phase I value at *p* < 0.05.

The WL%s at six-month follow-up were comparable among patients with and without SO (−10.49 ± 5.75 vs. −12.73 ± 4.30 Kg; *p* = 0.148) (Table 2, Figure 1). However, after more than 12 months, patients who had SO at baseline were able to maintain a lower WL% when compared to those without SO (−7.34 ± 6.29% vs. −11.43 ± 4.31%; *p* = 0.024) (Table 2, Figure 1). 

A comparison within the same subgroup across the treatment indicated that patients with SO showed a significant increase in body weight (87.58 ± 12.92 vs. 91.43 ± 15.62 Kg; *p* = 0.01) and a significant decrease in their WL% (−10.98 ± 5.79% vs. −7.34 ± 6.29%, *p* = 0.01) after more than 12 months, by comparison with the six-month follow-up, indicating a significant body weight regain after the WL phase. This was not evident in the group without SO, which showed no significant changes in body weight (73.18 ± 11.93 vs. 74.58 ± 10.77 Kg, *p* = 0.093) and WL% between the WL and the weight-maintenance phases (−13.27 ± 4.26% vs. −11.43 ± 4.31%, *p* = 0.060). 

In fact, partial correlation analysis revealed that only SO had a significant correlation with reduced WL% (*ρ* = −0.425, *p* = 0.009) while controlling for age, sex, and BMI. 

## 4. Discussion

The current study aimed to provide data on the relationship between SO and treatment outcomes, namely, the WL% among adults with obesity; one main finding was revealed.

The group of individuals with SO, when compared to those without SO, showed a comparable WL% at the six-month follow-up, which turned out to be significantly lower after a longer follow-up, indicating that SO is inversely associated with WL% after more than 12 months. This implies that despite the fact that patients with SO do not differ in terms of achieving similar rates of WL during the initial weight-management phase (i.e., six months), it seems that they (i.e., those with SO) face more difficulties in maintaining their WL over a longer period (i.e., at a follow-up after more than 12 months). 

To date, our study is one of the very few to report such a finding in the literature. Hence, it is difficult to compare it with previous studies conducted among this population, especially in terms of a longer follow-up (i.e., >12 months), and the use of lifestyle modification programs for weight management. To the best of our knowledge, one study did not observe any difference at three, six, and 12 months after bariatric surgery between SO and non-SO groups regarding weight-loss outcomes [18]. However, no further follow-up data were available. We speculate that one of the reasons behind our finding may be the lower energy expenditure (i.e., resting energy expenditure) and/or the more sedentary lifestyle evident among patients with SO, when compared to those without SO [14,16,30]. However, we believe that these metabolic and lifestyle disadvantages become more influent and play a determinant role in the late and advanced stages of weight-management programs, namely, during the maintenance phase (i.e., at a follow-up after more than 12 months). Therefore, future studies focusing on the mechanisms that prevent patients with SO from maintaining their WL in the long term are required in order to draw firm conclusions.

Our study has certain strengths. It is the first to assess the relationship between WL and SO among patients with obesity. Secondly, the longitudinal design and real-world setting of the study should both be considered strengths. On the other hand, the study has several limitations. First and foremost, data were obtained in a single unit by applying one treatment program, which meant that external validation was required. Secondly, the sample in this study only included patients who had undergone an outpatient WL program, therefore, our finding may not generalize to patients with obesity that seek other treatment modalities (bariatric surgery, drug interventions, etc.). Thirdly, we assessed body composition using an impedance analyzer, which, despite being validated, has still not been accepted as a gold-standard technique for patients with obesity [31]. Finally, due to limited sample size, these results are preliminary and need further replication in a larger population to provide firm conclusions. However, if confirmed, our finding may have relevant clinical implications for targeting patients with SO, who are more likely to face difficulties in maintaining the WL achieved during obesity treatment. Therefore, implementing additional strategies for this subgroup of patients [32,33,34] may be useful, especially during the maintenance phase. 

## 5. Conclusions

In this study, we provide evidence—even if is still considered preliminary and cannot be considered conclusive—that SO seems to be a factor that may affect the ability of patients with obesity to maintain their WL in the long term (i.e., after more than 12 months) in an outpatient, weight management, clinical setting. Certainly, due to the small study population, this finding should be interpreted with caution, since it still needs to be confirmed through replication in larger samples. However, should this finding be proven to be correct, this emphasizes the importance of developing specific strategies for these patients (i.e., those with SO) on weight-management programs, especially during the later stages. 

## Figures and Tables

**Table 1:** Sociodemographic and anthropometric characteristics of the study population at baseline *.

Variables	Total *n* = 46	SO *n* = 21	NSO *n* = 25	Significance
Age	44.25 (15.85)	46.43 (17.01)	42.42 (14.91)	*p* = 0.404
Sex				X^2^ = 0.097; *p* = 0.755
Males	10 (21.7)	5 (23.8)	5 (20.0)	
Females	36 (78.3)	16 (76.2)	20 (80.0)	
Level of education				X^2^ = 0.218; *p* = 0.641
Lower education	29 (63.0)	14 (66.7)	15 (60.0)	
Higher education	17 (37.0)	7 (33.33)	10 (40.0)	
Marital status				X^2^ = 0.017; *p* = 0.895
Not Married	18 (39.1)	8 (38.1)	10 (40.0)	
Married	28 (60.9)	13 (61.9)	15 (60.0)	
Employment				X^2^ = 0.017; *p* = 0.895
Unemployed	29 (63.0)	14 (66.7)	15 (60.0)	
Employed	17 (37.0)	7 (33.3)	10 (40.0)	
Weight (Kg)	92.09 (15.04)	97.77 (14.88)	87.32 (13.70)	*p* = 0.018
Height (cm)	160.45 (8.50)	159.07 (7.74)	161.61 (9.07)	*p* = 0.312
BF (Kg)	38.04 (11.50)	45.04 (12.99)	32.16 (5.34)	*p* < 0.0001
BF%	40.52 (6.29)	44.63 (6.08)	37.07 (4.02)	*p* < 0.0001
BMI (Kg/m^2^)	35.71 (4.84)	38.64 (4.55)	33.25 (3.59)	*p* < 0.0001
WC (cm)	110.80 (12.36)	115.31 (12.89)	107.14 (10.87)	*p* = 0.047
Obesity comorbidities				X^2^ = 0.031; *p* = 0.860
No	39 (86.7)	18 (85.7)	21 (87.5)	
Yes	6 (13.3)	3 (14.3)	3 (12.5)	

* The Values are Mean (SD) for continuous variables and (*n*%) for categorical variables; SO = sarcopenic obesity; BF = body fat; BMI = body mass index; WC = waist circumference.

**Table 2:** Body weight, weight loss, and weight-loss percentage at six-month and >12-months follow-up.

Variables	6 Months				>12 Months			
	Total *n* = 46	SO *n* = 21	Non-SO *n* = 25	*p*-Value	Total *n* = 40	SO *n* = 19	NSO *n* = 21	*p*-Value
Body weight (Kg)	81.38 (14.43)	87.24 (12.36)	76.45 (14.41)	*p* = 0.009	82.59 (15.64)	91.43 (15.62)	74.58 (10.77)	<0.0001
Weight loss (Kg)	−10.72 (5.16)	−10.53 (6.68)	−10.87 (3.56)	*p* = 0.834	−8.49 (5.19)	−7.24 (6.24)	−9.60 (3.84)	0.166
Weight loss (%)	−11.71 (5.08)	−10.49 (5.75)	−12.73 (4.30)	*p* = 0.148	−9.49 (5.66)	−7.34 (6.29)	−11.43 (4.31)	0.024

SO = sarcopenic obesity.

## Data Availability

Are available from the corresponding author on reasonable request.
